# Effect of progesterone concentration on hCG trigger day on clinical outcomes after high-quality single blastocyst transfer in GnRH antagonist cycles

**DOI:** 10.3389/fmed.2024.1443624

**Published:** 2024-10-09

**Authors:** Nan Jia, Jianing Xu, Bingbing Song, Haoying Hao, Meng Li, Cuilian Zhang, Shaodi Zhang

**Affiliations:** ^1^Reproductive Medicine Center, Henan Provincial People’s Hospital, ZhengZhou, China; ^2^Reproductive Medicine Center, People’s Hospital of Zhengzhou University, ZhengZhou, China

**Keywords:** trigger-day progesterone, GnRH antagonist cycle, high-quality single blastocyst, fresh cycle, frozen–thawed cycle, clinical pregnancy

## Abstract

**Objective:**

To investigate whether progesterone levels on the human chorionic gonadotropin (hCG) trigger day are associated with clinical outcomes in fresh cycles and the first frozen–thawed cycles (the freeze-all strategy) following the transfer of a high-quality single blastocyst.

**Methods:**

This single-center retrospective analysis was conducted on patients undergoing *in vitro* fertilization with the gonadotropin-releasing hormone (GnRH) antagonist protocol from January 2017 to December 2023. The study included the first and second oocyte retrieval cycles with progesterone levels ≤2 ng/ml on hCG trigger day. Clinical pregnancy rates and early miscarriage rates were compared among groups using curve fitting, threshold effect analysis, and multivariable regression.

**Results:**

When progesterone levels were between 1 and 2 ng/ml, the pregnancy rate in fresh cycles was only 51% of that in cycles with progesterone levels ≤1 ng/ml (95% CI: 0.33, 0.79, *p* = 0.0028). And the pregnancy rate decreased by 25% (95% CI: 0.51, 1.09) for frozen cycles, although there was no statistically significant (*p* = 0.1273). When cycle types were used as a binary variable in multivariate regression analysis, it was found that the clinical pregnancy rate in frozen cycles was 1.84 times higher than in fresh cycles (OR = 1.84, 95% CI: 1.38–2.47). For progesterone levels between 1 and 2 ng/ml, the clinical pregnancy rate in frozen cycles was 2.90 times that of fresh cycles (OR = 2.90, 95% CI: 1.59, 5.29, *p* = 0.0015). Progesterone levels on hCG day had no impact on the clinical pregnancy rate in thaw cycles, nor did they affect miscarriage rates in fresh or thaw cycles (*p* > 0.05).

**Conclusion:**

When progesterone levels on hCG trigger day were between 1 and 2 ng/ml, the clinical pregnancy rate for frozen–thawed cycles of high-quality single blastocyst transfer using an GnRH antagonist protocol significantly surpasses that of fresh cycles, thus elective frozen embryo transfer after the freeze-all strategy is recommended.

## Introduction

Under physiological conditions, an increase in progesterone typically occurs after ovulation, transforming the endometrium from the proliferative to the secretory phase, thereby facilitating embryo implantation. However, in controlled ovarian stimulation cycles used in assisted reproductive technologies (ART), factors such as the use of gonadotropins and the development of multiple follicles can lead to a premature rise in serum progesterone levels. Reports indicate that the incidence of premature progesterone elevation is about 13 to 46% (1) in the GnRH agonist protocols and 9 to 38% (2, 3) in antagonist protocols. Premature elevations in serum progesterone levels may affect oocyte and embryo quality (4, 5), as well as endometrial receptivity (6, 7). Some studies suggest that elevated serum progesterone levels have a greater impact on clinical pregnancy outcomes following cleavage-stage embryo transfers (6, 8), while findings are inconsistent regarding blastocyst transfers (2, 9).

Research on the impact of elevated serum progesterone on clinical outcomes after embryo transfer has been ongoing for some time and remains controversial. This is mainly due to variations across studies in ovarian stimulation protocols, measurement methods and units (10) (ng/ml, nmol/L), timing of measurement (hCG trigger day, day of egg retrieval (11), day of transfer (12)], and stages of embryo transferred (cleavage stage, blastocyst stage), leading to reports of different optimal serum progesterone ranges.

Many researchers have set the threshold for elevated serum progesterone at 1.5 ng/ml on hCG trigger day (11, 13). However, recent findings indicate that even mild increases (3, 14) in progesterone levels (1–1.5 ng/ml) can adversely affect clinical outcomes. Before applying the threshold values suggested in these reports to clinical practice, further researches are needed to assess the reproducibility of these conclusions. A study by Jianing Xu et al. at our center (15) compared the outcomes of transferring at least one high-quality cleavage-stage embryo or blastocyst in fresh cycles using an antagonist protocol, revealing a curvilinear relationship between serum progesterone levels on hCG trigger day and pregnancy outcomes, with an optimal threshold of 0.80 ng/ml. Based on this, we further compared the clinical pregnancy outcomes of different progesterone levels in fresh and frozen–thawed cycles to explore the progesterone cutoff points for fresh transfers and the first frozen–thawed cycles (the freeze-all strategy) in antagonist protocols, aiming to develop appropriate embryo transfer strategies.

Our study compared the clinical outcomes of fresh embryo transfers and first frozen embryo transfers (FET) with a freeze-all strategy on different progesterone levels on hCG trigger day. To minimize the influence of various factors, strict criteria were applied regarding patient age, endometrial thickness, blastocyst quality, and the ovarian stimulation protocol. The goal was to identify the optimal serum progesterone level for the transfer of a single high-quality blastocyst in an antagonist protocol and to establish a threshold level of serum progesterone on the day of hCG for the freeze-all strategy.

## Materials and methods

### Study population

This retrospective analysis included 1,390 cycles from patients who underwent *in vitro* fertilization (IVF) or intracytoplasmic sperm injection (ICSI) at the Reproductive Medicine Center of Henan Provincial People’s Hospital from January 2017 to December 2023. Due to the center’s policy of implementing a freeze-all strategy for fresh cycles with progesterone levels >2 ng/ml on the day of hCG, this study only included patients with progesterone levels ≤2 ng/ml on hCG trigger day.

### Inclusion criteria

- Female age ≤ 40 years.
- Use of the GnRH antagonist protocol for ovarian stimulation.
- Within the first or second oocyte retrieval cycles.
- The first FET cycle following a freeze-all strategy.
- Endometrial thickness ≥ 8 mm on the day of hCG or on the embryo transfer day.
- Transfer of a single high-quality blastocyst.

### Exclusion criteria

- Chromosomal abnormalities in either partner.
- Unresolved uterine issues such as adhesions, submucosal fibroids, or hydrosalpinx.
- Congenital or acquired uterine anomalies, including septate, saddle-shaped, unicornuate, or bicornuate uterus.
- History of recurrent miscarriage or adverse pregnancy history.
- Cycles using donor sperm or frozen eggs.
- Missing critical information.

### Research methods

1. Ovarian Stimulation Protocol: A flexible GnRH antagonist protocol was used. Gonadotropin (Gn) administration began on days 2–3 of the menstrual cycle. The antagonist (Cetrotide, 0.25 mg per dose, Merck Serono, Germany) was added once the estrogen level exceeded 600 pg/ml, the diameter of the dominant follicle reached 12–14 mm, or the luteinizing hormone (LH) level was twice the baseline. Oocyte retrieval occurred 34–36 h after triggering. As soon as two or three follicles of ≥17 mm were observed, final oocyte maturation was triggered with 5,000 or 10,000 IU of urinary hCG (Lishenbao, Livzon Pharmaceutical Co., Ltd.). For further details, see reference (15).
2. Progesterone Measurement: Blood samples were collected between 7:00–9:00 AM on the day of hCG administration for routine measurement of estrogen levels (E_2_), LH, and progesterone (P). Progesterone was measured using an electrochemiluminescence method (cobas 602, Roche Diagnostics, Germany), with a detection limit of 0.05 ng/ml. In addition to regular internal quality controls, calibration of the assay was promptly conducted whenever results exceeded normal ranges or when a new batch of reagents was introduced.
3. Embryo Grading and Transfer: High-quality blastocysts (16) were defined as those graded ≥3BB. Poor-quality blastocysts were considered surplus. Fresh embryo transfer criteria included progesterone levels <2 ng/ml on the day of hCG, endometrial thickness ≥ 8 mm with uniform echogenicity, absence of ovarian hyperstimulation syndrome tendencies, infection, or other special medical histories. The first thaw cycle after a freeze-all strategy was included for frozen transfers. Embryos were thawed using a vitrification and rapid warming method (Vitrification Kit, Thawing Kit, Kitazato, Tokyo, Japan). Endometrial preparation protocols included natural cycles and hormone replaced cycles (HRT) with endometrial thickness reaching at least 8 mm before adding progesterone for transformation. Subsequently, embryo transfer was performed. Progesterone support continued in single blastocyst transfers, and the luteal support was continued until 10 weeks if clinical pregnancy was achieved.
4. Observation Indicators: Clinical pregnancy was confirmed by the presence of intrauterine or ectopic gestational sacs on transvaginal ultrasound performed at 28 and 35 days post-transfer. Early miscarriage was defined as a miscarriage or arrested embryonic development occurring before 12 weeks. Clinical pregnancy rate = number of clinical pregnancy cycles / number of transfer cycles × 100%; early miscarriage rate = number of early miscarriage cycles / number of clinical pregnancy cycles × 100%.

### Statistical analysis

Statistical analyses were performed using EmpowerStats, a tool based on the R software platform. Data normally distributed were expressed as mean ± standard deviation (Mean ± SD), while non-normally distributed continuous variables were presented as medians (25th percentile, 75th percentile). Categorical variables were expressed as percentages (%). Comparisons between groups were conducted using the t-test, Chi-square test, or Fisher’s exact test as appropriate.

Univariate analyses were performed to identify factors potentially influencing clinical pregnancy outcomes. Covariates were examined and selected based on these analyses. Factors that showed statistically significant differences were then adjusted for in multivariate logistic regression analyses. Additionally, fitting curves were drawn to perform threshold effect analysis, and based on these results, the progesterone levels on the day of hCG were stratified for further analysis. A *p*-value <0.05 was considered statistically significant.

## Results

### General characteristics of patients

This study included a total of 1,390 cycles involving the transfer of a single high-quality blastocyst using an GnRH antagonist protocol, with 735 cycles in fresh transfers and 655 in frozen–thawed cycles. Baseline characteristics for both groups included female age, body mass index (BMI), duration of infertility, type of infertility, baseline FSH levels, antral follicle count (AFC), fertilization type, gonadotropins (Gn) dosage, Gn duration, E_2_ concentration on hCG trigger day, total number of oocytes retrieved, and endometrial thickness (EMT).

The clinical pregnancy rate for single high-quality blastocyst transfers in fresh cycles was 60.27%, and the early miscarriage rate was 10.16%. In contrast, the clinical pregnancy rate for frozen–thawed cycles was 70.84%, with an early miscarriage rate of 12.07%. Overall, the clinical pregnancy rate was significantly higher in frozen–thawed cycles compared to fresh cycles (*p* < 0.001), while the early miscarriage rates showed no significant difference (*p* = 0.360; Supplementary Table S1).

When stratifying progesterone levels into ≤1 and 1–2 ng/ml groups, a more pronounced difference in clinical pregnancy rates was observed in the latter group. The clinical pregnancy rate in the fresh cycles was 68.22%, significantly higher than in frozen–thawed cycles, which had a pregnancy rate of 44.35% (*p* < 0.001). Further details are presented in Table 1.

**Table 1:** Comparison of general patient characteristics.

	Progesterone ≤1 ng/ml			Progesterone =1–2 ng/ml		
	Fresh cycles	Frozen–thawed cycles	*P*-value	Fresh cycles	Frozen–thawed cycles	*P*-value
N	620	419		115	236	
Female age	31.74 ± 4.03	31.26 ± 3.90	0.061	31.14 ± 3.73	30.57 ± 4.03	0.206
Female BMI	23.08 ± 3.62	23.27 ± 3.89	0.424	22.15 ± 3.59	22.91 ± 3.73	0.069
Duration of infertility (year)	3.00 (1.45–5.00)	3.00 (1.50–5.00)	0.522	3.00 (2.00–4.00)	3.00 (2.00–4.70)	0.869
Infertility type			0.002			0.285
Secondary Infertility	377 (60.81%)	215 (51.31%)		64 (55.65%)	117 (49.58%)	
Primary Infertility	243 (39.19%)	204 (48.69%)		51 (44.35%)	119 (50.42%)	
Fertilization type			<0.001			0.101
IVF	508 (84.11%)	317 (75.66%)		88 (83.02%)	177 (75.00%)	
ICSI	96 (15.89%)	102 (24.34%)		18 (16.98%)	59 (25.00%)	
Basal FSH concentration (mIU/ml)	7.51 ± 2.91	6.44 ± 2.15	<0.001	7.38 ± 2.67	6.03 ± 1.66	<0.001
AFC	10.00 (7.00–15.00)	16.00 (10.00–22.00)	<0.001	9.00 (7.00–12.50)	17.00 (11.50–23.00)	<0.001
Gn dosage (IU)	2189.62 ± 765.65	1931.32 ± 861.00	<0.001	2425.00 ± 801.40	2008.17 ± 716.64	<0.001
Gn duration (day)	9.38 ± 1.75	9.18 ± 2.11	0.104	9.98 ± 1.65	9.47 ± 1.73	0.009
*p* concentration on hCG trigger day (ng/ml)	0.58 ± 0.23	0.59 ± 0.23	0.499	1.21 ± 0.21	1.36 ± 0.26	<0.001
E_2_ concentration on hCG trigger day (pg/ml)	1839.50 (1214.25–2631.00)	2054.50 (1276.75–3000.00)	0.009	2816.50 (1725.75–4121.50)	2356.00 (1596.50–3890.00)	0.092
No. of retrieved oocytes	9.00 (6.00–12.00)	13.00 (9.00–18.00)	<0.001	11.00 (8.00–14.00)	16.00 (11.00–21.00)	<0.001
No. of available embryos	6.28 ± 2.80	8.00 (5.00–11.00)	<0.001	7.09 ± 2.95	8.00 (6.00–12.00)	<0.001
Endometrial thickness (mm)	11.25 ± 2.16	9.96 ± 1.63	<0.001	11.11 ± 1.95	9.85 ± 1.53	<0.001
Clinical pregnancy (%)	392 (63.23%)	303 (72.32%)	0.002	51 (44.35%)	161 (68.22%)	<0.001
Early miscarriage (%)	40 (10.20%)	38 (12.54%)	0.333	5 (9.80%)	18 (11.18%)	0.783

### Progesterone level fitting curves on the day of hCG

**Figure 1:**
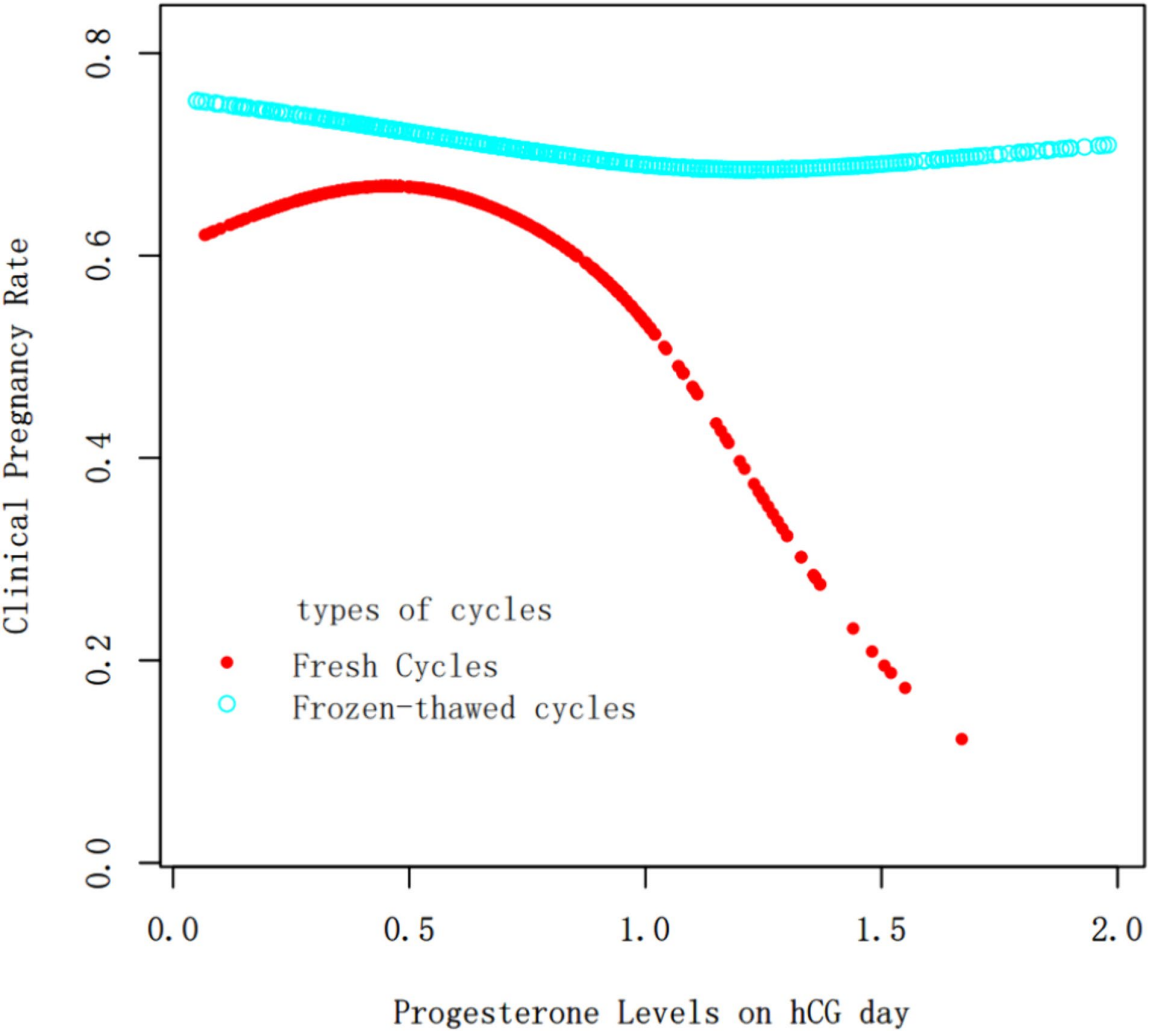
Fitting curve of clinical pregnancy outcomes and progesterone levels on hCG trigger day in fresh cycles vs. frozen–thawed cycles. The red and blue curves represent the fresh cycles and the frozen–thawed cycles, respectively. Adjust for age, BMI, infertility type, duration of infertility, basal FSH, AFC, Gn dosage, E_2_ concentration on hCG trigger day, No. of retrieved oocytes and EMT.

Progesterone Levels as a Continuous Variable: The relationship between progesterone levels and clinical pregnancy rates was analyzed through fitting curves for both fresh and frozen–thawed cycles (Figure 1). The curve for fresh transfers indicated that the clinical pregnancy rate remained relatively stable when progesterone levels were below 1 ng/ml, after which there was a sharp decline as progesterone levels increased. In contrast, for frozen–thawed cycles, the clinical pregnancy rate remained stable across different progesterone levels, showing no significant fluctuations and consistently higher rates compared to fresh transfers.

**Figure 2:**
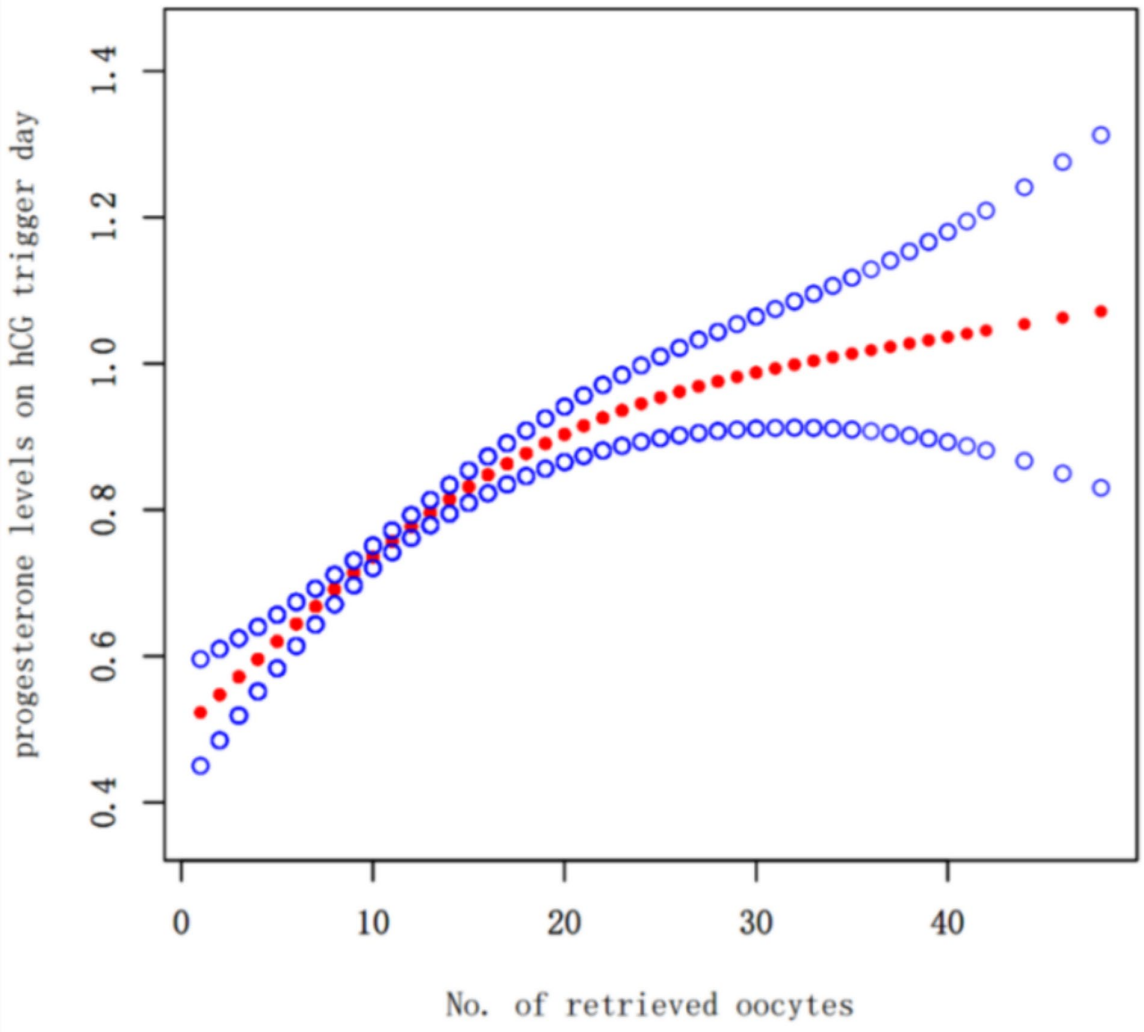
Fitting curve of no. of retrieved oocytes and progesterone levels on hCG trigger day the solid-dotted red line represents the smooth curve that fits between variables. Blue curves represent the upper and lower limits of the 95% CI. Adjusted for age, body mass index,the duration of infertility and infertility type.

Oocyte Yield as a Continuous Variable: A fitting curve was plotted between the number of oocytes retrieved and progesterone levels on hCG trigger day (Figure 2). The analysis, including a threshold effect, indicated that progesterone levels continuously increased with the number of oocytes retrieved, with the rate of increase slowing down around the retrieval of approximately 10 oocytes.

### Influence of progesterone levels on hCG trigger day on clinical outcomes in fresh and thaw transfer cycles

Using covariate screening aligned with clinical realities, factors such as female age, BMI, duration of infertility, type of infertility, basal FSH, AFC, Gn dosage, E_2_ levels on hCG trigger day, total oocytes retrieved, and EMT were adjusted in the multivariate regression analysis. A threshold effect analysis of progesterone levels on the day of hCG and clinical pregnancy rates in fresh cycles identified a breakpoint at 0.94 with a log-likelihood ratio test value of 0.036. Based on previous studies and clinical practices, the threshold was set at 1 ng/ml.

#### Multivariate regression analysis results

When analyzing progesterone levels on hCG day as a continuous variable in multivariate regression, the results showed that as progesterone levels increased, the clinical pregnancy rate in fresh cycles decreased (OR = 0.53, 95% CI: 0.32, 0.87), while there was no significant change in frozen cycles (OR = 0.88, 95% CI: 0.60, 1.27). This was consistent with the results of the fitting curve.

When progesterone levels were treated as a binary variable in multivariate regression, the clinical pregnancy rate was significantly lower for progesterone levels between 1 and 2 ng/ml compared to levels ≤1 ng/ml (OR = 0.51, 95% CI: 0.33, 0.79), with no significant change in frozen cycles (OR = 0.75, 95% CI: 0.51, 1.09).

Progesterone levels had no impact on miscarriage rates in fresh or thaw cycles (*p* > 0.05).

For a detailed presentation, see Table 2.

**Table 2:** Multivariate regression analysis of progesterone levels on hCG trigger day with clinical pregnancy outcomes in fresh and frozen–thawed cycles.

	Fresh cycles			Frozen–thawed cycles		
	OR	95%CI	*p*-value	OR	95%CI	*p*-value
Clinical pregnancy	0.53	0.32, 0.87	0.0114	0.88	0.60, 1.27	0.4851
*p* ≤ 1 ng/ml	Reference			Reference		
*p* = 1-2 ng/ml	0.51	0.33, 0.79	0.0028	0.75	0.51, 1.09	0.1273
Early miscarriage	0.52	0.16, 1.77	0.2992	1.21	0.64, 2.28	0.5569
*p* ≤ 1 ng/ml	Reference			Reference		
*p* = 1-2 ng/ml	1.3	0.44, 3.84	0.6373	1.05	0.55, 2.00	0.8882

Adjust for: Female age; Female BMI; Infertility type; Duration of infertility; Basal FSH; AFC; Gn dosage; E_2_ concentration on hCG trigger day; No. of retrieved oocytes; EMT.

### Comparison of clinical pregnancy outcomes between fresh and frozen cycles at different progesterone levels

Multivariate regression analysis, using cycle type (either fresh or frozen) as the exposure variable, showed that the clinical pregnancy rate for frozen cycles was 1.84 times that of fresh cycles (OR = 1.84, 95% CI: 1.38–2.47), with no statistically significant difference in miscarriage rates (*p* > 0.05). Stratified multivariate regression analysis indicated that when progesterone levels were ≤1 ng/ml, the clinical pregnancy rate for frozen transfer cycles was 1.67 times that of fresh transfer cycles (OR = 1.67, 95% CI: 1.22–2.29, *p* = 0.0005). As progesterone levels increased, i.e., between 1 ~ 2 ng/ml, the clinical pregnancy rate for frozen transfer cycles was 2.90 times that of fresh transfer cycles (OR = 2.90, 95% CI: 1.59–5.29, *p* = 0.0005). See Table 3 for details.

**Table 3:** Multivariate regression results for progesterone levels on hCG day.

	P		*p* ≤ 1 ng/ml		*p* = 1-2 ng/ml	
	OR (95%CI)	*p*-value	OR (95%CI)	*p*-value	OR (95%CI)	*p*-value
Clinical pregnancy						
Fresh cycles	Reference		Reference		Reference	
Frozen–thawed cycles	1.84 (1.38–2.47)	<0.0001	1.67 (1.22, 2.29)	0.0015	2.90 (1.59, 5.29)	0.0005
Early miscarriage						
Fresh cycles	Reference		Reference		Reference	
Frozen–thawed cycles	1.04 (0.61,1.78)	0.8905	1.18 (0.67, 2.08)	0.5564	1.96 (0.48, 8.08)	0.351

Adjust for: Female age; Female BMI; Infertility type; Duration of infertility; Basal FSH; AFC; Gn dosage; E_2_ concentration on hCG trigger day; No. of retrieved oocytes; EMT.

## Discussion

This study compared fresh cycles using an GnRH antagonist protocol with the first frozen–thawed cycles (the freeze-all strategy) following the transfer of a high-quality single blastocyst. The results indicated that the overall clinical pregnancy rate for frozen cycles was higher than for fresh cycles.

Based on the fitted curves and threshold effect analysis combined with clinical practice, the progesterone level on hCG trigger day was set at a threshold of 1 ng/ml. Stratified multivariate regression analysis showed that at progesterone levels of 1–2 ng/ml on hCG day, the clinical pregnancy rate for frozen transfer cycles was 2.90 times that of fresh transfer cycles, with no statistically significant difference in miscarriage rates. Therefore, we believe that when progesterone levels on hCG day exceed 1 ng/ml, opting for a freeze-all strategy and thawing cycle is advisable.

Additionally, the number of oocytes retrieved was plotted against progesterone levels to generate a fitting curve. The results showed that progesterone levels increased with the number of oocytes retrieved. The combined threshold effect analysis indicated that at around 10 retrieved oocytes, the increase in progesterone levels began to plateau, suggesting that in GnRH antagonist protocols, the increase in progesterone levels on hCG day is related to the number of oocytes retrieved.

With the use of ART, numerous studies have focused on how to improve clinical pregnancy and live birth rates. Among these, the impact of progesterone levels on hCG trigger day on embryos, endometrium, and clinical outcomes has been a significant area of research. However, the optimal serum progesterone level for fresh transfer or complete embryo freezing remains a contentious issue. Recent studies suggest that elevated progesterone levels on hCG day (>1.57 ng/ml) in fresh transfer cycles are negatively correlated with live birth rates (17), and most scholars believe that progesterone levels <1.5 ng/ml do not affect the outcomes of fresh transfer cycles (18). A prospective cohort study from Brazil (19) used a serum progesterone level of 1.5 ng/ml as a threshold; levels above this threshold prompted complete embryo freezing, while lower levels allowed for fresh transfers. Comparing fresh cycle transfers of cleavage stage embryos using antagonist protocols with frozen–thawed transfers, the study found significantly better clinical pregnancy outcomes in frozen cycles. Thus, the study suggests that even when serum progesterone levels are ≤1.5 ng/ml, endometrial receptivity may be compromised due to controlled ovarian stimulation, and adopting a complete embryo freezing strategy might improve clinical outcomes.

There has always been controversy regarding the threshold of progesterone levels on hCG day that affects the clinical outcomes of fresh embryo transfers. Contrary to the thresholds in earlier studies, recent research suggests that progesterone levels above 1 ng/ml can impact the clinical outcomes of fresh cycles. For example, a 2022 study (14) showed that even a slight increase in serum progesterone levels, between 1.0 to 1.5 ng/ml, affects the clinical pregnancy outcomes of single blastocyst transfers in a long protocol with early follicular phase in fresh cycles. Zhao et al. found that in the antagonist protocol (3), the group with slightly elevated progesterone levels (1.0 to 1.5 ng/ml) on hCG day had significantly lower clinical pregnancy rates in fresh cleavage-stage embryo transfer cycles compared to the normal progesterone group. Additionally, an Italian single-center retrospective cohort study (20) found that in a subgroup with good prognosis cleavage-stage embryo transfers, the clinical pregnancy and live birth rates began to progressively decline when progesterone levels exceeded 1 ng/ml. These findings are consistent with our results, which show that pregnancy rates for high-quality single blastocyst transfers in antagonist protocols are only 51% of those with progesterone levels ≤1 ng/ml when progesterone levels range between 1 and 2 ng/ml.

Embryo transfer strategies should not only focus on clinical pregnancy outcomes but also consider the safety of mothers and infants during pregnancy and the perinatal period. A 2018 meta-analysis (21) showed that obstetric complications, including gestational hypertension, preeclampsia, and placenta accreta, occur at significantly lower rates in fresh embryo transfers compared to frozen ones. Hence, when progesterone levels are ≤1 ng/ml, the difference in clinical pregnancy rates between fresh and thawed cycles is minimal, allowing for fresh embryo transfers to shorten the time to live birth and reduce the incidence of conditions such as preeclampsia (22) and macrosomia (23). Even though freezing technology, particularly vitrification, has matured with embryo survival and total live birth rates reaching over 95% or even higher, and the use of full embryo freezing strategies is becoming more common, we must still cautiously consider full embryo freezing strategies from the perspective of maternal and perinatal safety.

A study by Masami Abe et al. that included various ovarian stimulation protocols (including antagonist protocols, long protocols, and mild stimulation protocols) suggested (24) that the clinical pregnancy and live birth rates of blastocyst thawing and transfer cycles are higher than those of fresh transfer cycles. However, in cases where the progesterone level is <1.0 ng/ml, the clinical pregnancy rates of fresh blastocyst transfers are the same as those of thawed cycles, which slightly differs from our results. This study did not specify constraints on the quality of blastocysts, the number of embryos transferred, or the endometrial thickness, which are crucial factors affecting clinical pregnancy outcomes. Our study only included single high-quality blastocyst transfers in antagonist protocols with an endometrial thickness of ≥8 mm to minimize confounding factors. The reasons for better clinical pregnancy outcomes in single high-quality blastocyst thawing cycles compared to fresh cycles include the following (23): thawing cycles avoid the impact of supraphysiological hormone levels caused by stimulation on endometrial receptivity, providing a more favorable uterine microenvironment than fresh transfers; and thawed blastocysts are transferred on the 5th day after endometrial transformation, which offers better synchrony and is closer to the natural implantation process.

Furthermore, our baseline data show that AFC and the number of oocytes retrieved in the thawing cycle group are statistically significantly higher than those in the fresh transfer group. To analyze the reasons, we created a fitting curve of the number of oocytes retrieved and progesterone levels on hCG trigger day, showing that as the number of oocytes increases, so does the progesterone level. In clinical practice, to avoid ovarian hyperstimulation syndrome or high progesterone levels on hCG trigger day leading to decreased clinical outcomes, a freeze-all strategy is adopted; thus, the thawing cycle group has a higher average number of eggs retrieved and ovarian reserve indicators (AFC) than the fresh transfer group. Additionally, patients with a higher number of oocytes often have elevated progesterone levels, so in our data, the overall number of oocytes retrieved and progesterone levels are higher in the thawing cycle than in the fresh cycle. Oocytes retrieval numbers and ovarian reserve indicators affect the number of usable embryos and cumulative live birth rates, but our study only analyzed the clinical pregnancy rates of a single transfer cycle and limited the quality and number of embryos transferred to high-quality single blastocyst transfers. Moreover, there were no statistically significant differences in key indicators affecting clinical pregnancy outcomes, such as female age (25) and BMI (26); thus, the differences in these indicators do not affect our study results. Our previous research on the impact of endometrial thickness on clinical outcomes suggested that the best clinical outcomes occur when the endometrial thickness is between 8.7–14.5 mm (27). In this study, although the endometrial thickness during the thawing cycle was significantly lower than during the fresh cycle, it was still within the 8.7–14.5 mm range. Even without an advantage in endometrial thickness in the thawing cycle, the overall clinical pregnancy outcomes were still better than those of the fresh transfer cycle, and we adjusted for endometrial thickness in multivariable regression analysis and curve fitting to ensure the objectivity and reliability of our results.

The mechanism by which elevated progesterone levels prematurely affect the clinical pregnancy outcomes of fresh embryo transfer cycles remains controversial, particularly regarding the quality of oocytes and embryos (28). Studies have found (29) that even in patients with good embryo quality and prognosis, elevated progesterone levels on hCG day significantly reduce clinical pregnancy rates, suggesting that the negative effects of premature elevation of progesterone levels are independent of the stage of the transferred embryos, embryo quality, female age, or ovarian response levels. Our research also confirms that in young patients with a higher number of eggs retrieved, the clinical pregnancy rates of fresh cycle single high-quality blastocyst transfers are not as high as those in thawed cycles. Increasingly, studies suggest that elevated progesterone levels affect the gene expression and epigenetic changes in endometrial cells related to immune tolerance, disrupting the implantation window and negatively affecting its receptivity (28, 30). Researchers have analyzed the transcriptome of the endometrium during the implantation window (31) and found that when serum progesterone levels are high on hCG trigger day, the expression patterns of genes related to natural killer cell-mediated cytotoxic pathways in the endometrium are significantly different from those in normal endometrium.

This study included an GnRH antagonist protocol and only transferred a single high-quality blastocyst during the frozen–thawed cycle. Only the first transfer from a freeze-all strategy was included to prevent the best blastocyst from being transferred during the fresh cycle, thus maximizing the exclusion of embryo quality and the number of transferred embryos affecting the clinical pregnancy outcome. Strict inclusion and exclusion criteria were applied, and multiple variables were adjusted for multivariate regression analysis, combined with fitting curves and threshold effect analysis results, to perform stratified multivariate regression analysis, ensuring our conclusions are stable and reliable.

This study still has the limitations of a retrospective analysis, such as the first FET cycle after the freeze-all strategy, where the reasons for embryo freezing are mostly excessive follicular development combined with elevated progesterone levels, to avoid ovarian hyperstimulation syndrome. Due to the clinical workflow, AFC and the number of oocytes retrieved in the thaw cycle in this study were higher than those in the fresh blastocyst transfer cycle. However, since this study only compared the clinical outcomes of a single high-quality blastocyst transfer and not the cumulative live birth rate, and there were no statistically significant differences between the two groups in key indicators affecting the clinical outcomes of the single blastocyst transfer cycle such as patient age and embryo quality, it is considered that differences in AFC and the number of oocytes retrieved do not affect the results of this study. In the future, we can design prospective studies with strict inclusion and exclusion criteria to control baseline patient indicators as much as possible to analyze the impact of progesterone levels on the clinical outcomes. It should be noted that the conclusions of this study are only applicable to the GnRH antagonist protocol with high-quality single blastocyst transfer cycles and not suitable for other ovulation induction protocols or cleavage-stage embryo transfer cycles, and more studies and larger sample sizes are needed to validate the results of this study.

In summary, in the GnRH antagonist protocol, patients undergoing high-quality single blastocyst transfer in the frozen–thawed cycle can achieve better clinical pregnancy outcomes, especially when progesterone levels on hCG trigger day are >1 ng/ml, as thaw cycle transfer after the freeze-all strategy has a clear advantage over fresh cycle transfer. When progesterone levels on the day of hCG are ≤1 ng/ml, various factors such as the patient’s time cost, financial cost, time to achieve live birth, maternal and infant health, and patient’s preference should be comprehensively considered in the embryo transfer strategy.

## Data Availability

The raw data supporting the conclusions of this article will be made available by the authors, without undue reservation.

## References

[1] Wu Z Dong YH Ma Y Li Y Li L Lin N. Progesterone elevation on the day of hCG trigger has detrimental effect on live birth rate in low and intermediate ovarian responders, but not in high responders. Sci Rep. (2019) 9:5127. doi: 10.1038/s41598-019-41499-1, PMID: 30914679 PMC6435811

[2] Tokgoz VY Tekin AB. Serum progesterone level above 0.85 ng/mL and progesterone/estradiol ratio may be useful predictors for replacing cleavage-stage with blastocyst-stage embryo transfer in fresh IVF/ICSI cycles without premature progesterone elevation. Arch Gynecol Obstet. (2022) 305:1011–9. doi: 10.1007/s00404-021-06304-3, PMID: 34716819

[3] Zhao J Hao J Xu B Wang Y Li Y. Effect of slightly elevated progesterone on hCG trigger day on clinical pregnancy rate in GnRH-ant IVF/ICSI cycles. Reprod Health. (2022) 19:66. doi: 10.1186/s12978-022-01371-4 35287707 PMC8919624

[4] Racca A Santos-Ribeiro S de Munck N Mackens S Drakopoulos P Camus M. Impact of late-follicular phase elevated serum progesterone on cumulative live birth rates: is there a deleterious effect on embryo quality? Hum Reprod. (2018) 33:860–8. doi: 10.1093/humrep/dey031, PMID: 29481670

[5] Yadav A Noor N Mahey R Singh N Dwarakanathan V Malhotra N. Serum progesterone on the day of human chorionic gonadotropin (hCG) trigger as a predictor of in-vitro fertilization (IVF) outcome a retrospective analysis of seven years. JBRA Assist Reprod. (2023) 27:156–62. doi: 10.5935/1518-0557.20220023, PMID: 35916460 PMC10279434

[6] Yang SY Pang TS Li R Yang R Zhen X Chen X. The individualized choice of embryo transfer timing for patients with elevated serum progesterone level on the hCG day in IVF/ICSI cycles: a prospective randomized clinical study. Gynecol Endocrinol. (2015) 31:355–8. doi: 10.3109/09513590.2014.995620, PMID: 25558791

[7] Racca A Vanni VS Somigliana E Reschini M Viganò P Santos-Ribeiro S. Is a freeze-all policy the optimal solution to circumvent the effect of late follicular elevated progesterone? A multicentric matched-control retrospective study analysing cumulative live birth rate in 942 non-elective freeze-all cycles. Hum Reprod. (2021) 36:2463–72. doi: 10.1093/humrep/deab160, PMID: 34223890

[8] Li RR Dong YZ Guo YH Sun YP Su YC Chen F. Comparative study of pregnancy outcomes between day 3 embryo transfer and day 5 blastocyst transfer in patients with progesterone elevation. J Int Med Res. (2013) 41:1318–25. doi: 10.1177/0300060513489480, PMID: 23812114

[9] Demirel C Aydoğdu S Özdemir Aİ Keskin G Baştu E Buyru F. Blastocyst transfer does not improve cycle outcome as compared to D3 transfer in antagonist cycles with an elevated progesterone level on the day of hCG. J Turkish German Gynecol Assoc. (2017) 18:133–8. doi: 10.4274/jtgga.2017.0012, PMID: 28890427 PMC5590209

[10] Wu XD Mao YD Gao Y Qian X Wang W Ding W. Progesterone rise on hCG day is negatively correlated with IVF-ET outcomes in natural cycles. Clin Chim Acta. (2018) 478:194–9. doi: 10.1016/j.cca.2017.12.047, PMID: 29305844

[11] Roque Fernandez MA Alvarez Lleo C Gonzalez Mirasol E Resta Serra M Garcia Garrido C Sanchez Toledo M. Progesterone elevation on the day of oocyte retrieval and live birth rate after in vitro fertilisation treatment. J Obstet Gynaecol. (2022) 42:1396–400. doi: 10.1080/01443615.2021.1983780, PMID: 34907863

[12] Rozen G Rogers P Mizrachi Y Teh WT Parmar C Polyakov A. Serum progesterone concentration on the day of embryo transfer in stimulated cycles does not correlate with reproductive outcomes. Reprod Biomed Online. (2022) 45:1160–6. doi: 10.1016/j.rbmo.2022.07.015, PMID: 36137874

[13] Lee CI Chen HH Huang CC Lin PY Lee TH Lee MS. Early progesterone change associated with pregnancy outcome after fresh embryo transfer in assisted reproduction T echnology cycles with progesterone level of >1.5 ng/ml on human chorionic gonadotropin trigger day. Front Endocrinol (Lausanne). (2020) 11:653. doi: 10.3389/fendo.2020.00653, PMID: 33042015 PMC7522275

[14] Wei LL Zhao Y Xu CY Zhang C. Slightly elevated progesterone on hCG T rigger day has an impact on pregnancy outcomes of fresh single blastocyst T ransfer cycles under an early follicular phase prolonged protocol cycle. Int J Women's Health. (2022) 14:1761–8. doi: 10.2147/IJWH.S385362 36568124 PMC9784381

[15] Xu J Zhang C Wang S Zhang S. Impact of progesterone concentration on human chorionic gonadotropin trigger day on clinical outcomes with one top-quality cleavage-stage embryo or blastocyst transfer in fresh in vitro fertilization cycles. Front Endocrinol. (2023) 14:1085287. doi: 10.3389/fendo.2023.1085287 PMC10319152 37409225

[16] Hu KL Zheng X Hunt S Li X Li R Mol BW. Blastocyst quality and perinatal outcomes in women undergoing single blastocyst transfer in frozen cycles. Hum Reprod Open. (2021) 2021:hoab036. doi: 10.1093/hropen/hoab036, PMID: 35187269 PMC8849119

[17] Lepage J Keromnes SG Epelboin S Luton D Yazbeck C. Premature progesterone rise on day of hCG negatively correlated with live birth rate in IVF cycles: an analysis of 1022 cycles. J Gynecol Obstet Hum Reprod. (2019) 48:51–4. doi: 10.1016/j.jogoh.2018.05.005 29783037

[18] Venetis CA Kolibianakis EM Bosdou JK Lainas GT Sfontouris IA Tarlatzis BC. Estimating the net effect of progesterone elevation on the day of hCG on live birth rates after IVF:a cohort analysis of 3296 IVF cycles. Hum Reprod. (2015) 30:684–91. doi: 10.1093/humrep/deu362, PMID: 25586787

[19] Roque M Valle M Guimarães F Sampaio M Geber S. Freeze-all policy: fresh vs.frozen-thawed embryo transfer. Fertil Steril. (2015) 103:1190–3. doi: 10.1016/j.fertnstert.2015.01.045, PMID: 25747130

[20] de Cesare R Morenghi E Cirillo F Ronchetti C Canevisio V Persico P. The role of hCG triggering progesterone levels: a real-world retrospective cohort study of more than 8000 IVF/ICSI cycles. Front Endocrinol. (2020) 11:547684. doi: 10.3389/fendo.2020.547684, PMID: 33071968 PMC7538643

[21] Roque M Valle M Sampaio M Geber S. Obstetric outcomes after fresh versus frozen-thawed embryo transfers: a systematic review and meta-analysis. JBRA Assist Reprod. (2018) 22:253–60. doi: 10.5935/1518-0557.20180049, PMID: 29782139 PMC6106638

[22] Chen Z-J Shi Y Sun Y Zhang B Liang X Cao Y. Fresh versus frozen embryos for infertility in the polycystic ovary syndrome. N Engl J Med. (2016) 375:523–33. doi: 10.1056/NEJMoa1513873, PMID: 27509101

[23] Wei D Liu J-Y Sun Y Shi Y Zhang B Liu J-Q. Frozen versus fresh single blastocyst transfer in ovulatory women: a multicentre, randomised controlled trial. Lancet. (2019) 393:1310–8. doi: 10.1016/S0140-6736(18)32843-5, PMID: 30827784

[24] Abe M Yamamoto Y Noguchi H Tamura K Aoki H Takeda A. Is a freeze-all strategy necessary for all embryo transfers: fresh embryo transfer without progesterone elevation results in an equivalent pregnancy rate to cryopreserved embryo transfer. J Med Investig. (2022) 69:224–9. doi: 10.2152/jmi.69.224, PMID: 36244773

[25] Auctores Publishing LLC Naeiji Z Zademodares S Abbaspour M Anbarluei M Rahmati N. In-vitro fertilization outcome in patients with polycystic ovary syndrome: role of age and maternal body weight. Women Health Care Issues. (2021) 4:01–5. doi: 10.31579/2642-9756/056, PMID: 22698930

[26] Zheng D Wang Y Chen L Zeng L Li R. Association between body mass index and in vitro fertilization/intracytoplasmic sperm injection outcomes: an analysis of 15,124 normal ovarian responders in China. Chin Med J. (2024) 137:837–45. doi: 10.1097/CM9.0000000000002992, PMID: 38494342 PMC10997229

[27] Shaodi Z Qiuyuan L Yisha Y Cuilian Z. The effect of endometrial thickness on pregnancy outcomes of frozen-thawed embryo transfer cycles which underwent hormone replacement therapy. PLoS One. (2020) 15:e0239120. doi: 10.1371/journal.pone.0239120, PMID: 32970718 PMC7513995

[28] Jiang WH Li D Zhu LH Wang J Chen L Zhang N. Elevated serum progesterone levels on the hCG trigger day have a negative impact on the live birth rate in the first fresh IVF-ET cycle hCG. J Obstet Gynaecol. (2022) 42:3503–8. doi: 10.1080/01443615.2022.2151345, PMID: 36451550

[29] Hill MJ Greene Donald Royster MWH 4th Richter KS Levy G DeCherney AH Levens ED. Are good patient and embryo characteristics protective against the negative effect of elevated progesterone level on the day of oocyte maturation? Fertil Steril. (2015) 103:1477–1484.e5. doi: 10.1016/j.fertnstert.2015.02.038, PMID: 25881880 PMC6499929

[30] González-Foruria I Rodríguez I Martínez F Rodríguez-Purata J Montoya P Rodríguez D. Clinically significant intra-day variability of serum progesterone levels during the final day of oocyte maturation: a prospective study with repeated measurements. Hum Reprod. (2019) 34:1551–8. doi: 10.1093/humrep/dez091, PMID: 31334546

[31] Liu L Zhao LJ Li TC Zhu H Lin X Jin X. Comparison of progesterone measurement on day of, and day after, hCG administration in IVF-embryo transfer cycles. Reprod Biomed Online. (2015) 30:157–65. doi: 10.1016/j.rbmo.2014.10.017, PMID: 25530034

