# Supplementary material for: Effect of progesterone concentration on hCG trigger day on clinical outcomes after high-quality single blastocyst transfer in GnRH antagonist cycles
Source: Front Med (Lausanne). 2024 Oct 9;11:1443624. doi: 10.3389/fmed.2024.1443624 (PMC11514136; doi:10.3389/fmed.2024.1443624)
Supplement: Supplementary file 1 [file Table_1.docx]

Supplementary table 1

|  | Fresh cycles | Frozen-thawed cycles | P-value |
| --- | --- | --- | --- |
| Clinical pregnancy(%) | 443 (60.27%) | 464 (70.84%) | <0.001 |
| Early miscarriage(%) | 45 (10.16%) | 56 (12.07%) | 0.360 |
